# Oropouche Virus Isolation, Southeast Brazil

**DOI:** 10.3201/eid1110.050464

**Published:** 2005-10

**Authors:** Márcio Roberto Teixeira Nunes, Lívia Carício Martins, Sueli Guerreiro Rodrigues, Jannifer Oliveira Chiang, Raimunda do Socorro da Silva Azevedo, Amelia P.A. Travassos da Rosa, Pedro Fernando da Costa Vasconcelos

**Affiliations:** * Instituto Evandro Chagas, Belém, Pará, Brazil; † University of Texas Medical Branch, Galveston, Texas, USA

**Keywords:** Oropouche virus, Arinos, Southeast, Brazil, Callithrix sp., genotype, dispatch

## Abstract

An Oropouche virus strain was isolated from a novel host (*Callithrix* sp.) in Arinos, Minas Gerais State, southeastern Brazil. The virus was identified by complement fixation test and confirmed by reverse transcription–polymerase chain reaction. Phylogenetic analysis identified this strain as a genotype III isolate previously recognized only in Panama.

Oropouche virus (OROV) is one of the most important arthropodborne orthobunyaviruses (*Bunyaviridae*, *Orthobunyavirus*) (*1*) that infect humans; it causes an acute febrile illness called Oropouche fever (*2*). OROV was originally reported in Trinidad in 1955, when the prototype virus strain was isolated from the blood of a febrile patient and from a pool of *Coquillettidia venezuelensis* mosquitoes (*3*). In Brazil, OROV was initially described in 1960, when it was isolated from a sloth (*Bradypus tridactylus*) captured near a forest area during the construction of the Belém-Brasilia highway, and from a pool of *Ochlerotatus serratus* mosquitoes captured nearby (*4*). More than one-half million persons have been infected with OROV, which makes this virus a public health threat in tropical and subtropical areas of Central and South America (*5*). OROV genome consists of 3 partite, single-stranded, negative-sense RNAs, named large (L), medium (M), and small (S) RNA. These RNAs are predicted to encode a large protein (L: polymerase activity), viral surface glycoproteins (Gc and Gn), and nonstructural NSM protein, as well as both nucleocapsid (N) and NSS proteins (*1*). Complete nucleotide sequences have been determined for all 3 RNA segments (*6**–**8*), and previous studies of the molecular biology of the N gene (SRNA) of 28 different OROV strains indicated the existence of 3 genotypes, designated I, II, and III (*6*).

## The Study

**Figure 1:**
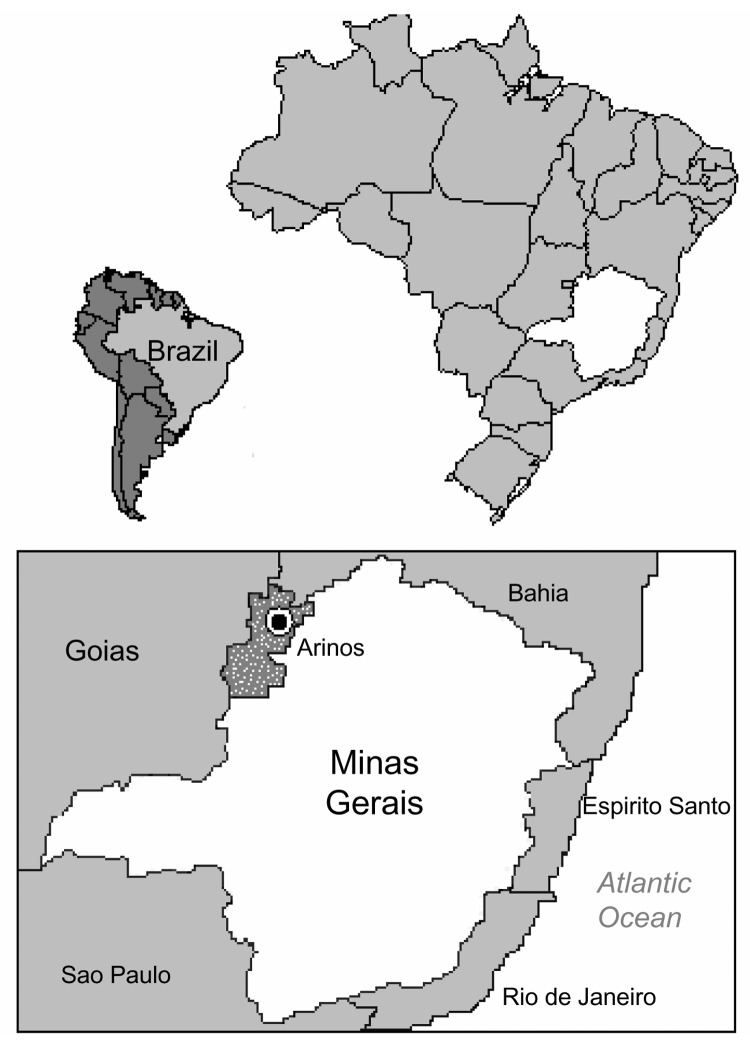
Map of the Arinos region, where the strain BeAN 626998 was isolated from a sylvatic monkey of the genus Callithrix.

A field study was conducted in the Arinos region, Minas Gerais State, within the Grande Sertão Veredas National Park, in February 2000. The park is located 90 km from Arinos city (15°54´S, 46°W) (Figure 1). The Arinos region is part of the epizootic area of sylvatic yellow fever virus transmission, as previously reported (*9*).

Using a biosafety cabinet class II B-2, we prepared suspensions of viscera (liver, spleen, and kidney) obtained from the monkey and then injected suckling mice by the intracerebral route. Animals were observed daily and were immediately collected when they died or showed signs of disease.

The virus was identified by using the complement fixation test (CF), performed according to a described technique (*10*). The BeAn 626990 antigen was tested against different mice immune ascitic fluids (MIAFs) prepared for a large number of arboviruses circulating in Brazil.

Molecular studies were conducted to confirm the serologic results. Viral RNA was extracted by using the Trizol reagent (Invitrogen, Carlsbad, CA, USA) technique as described elsewhere, and the entire SRNA segment was amplified, applying a 1-step reverse transcription–polymerase chain reaction (RT-PCR) assay using the primers ORO N5 (5´ AAAGAGGATCCAATAATGTCAGAGTT CATTT 3´), ORO N3 (5´ GTG AAT TCC ACT ATA TGC CAA TTC CGA ATT 3´), ORO 1A (5´ AGTAGTGTACTCCACTAT 3´) (*6*), ORONR 271 (5´ CGACTGGAACTGTGGGAAAT 3´), OROF593 (5´ AAGTCCTCCGGCAGAGGTAT 3´), and ORO2S (5´ AGT AGT GTG GCT CCA CAT 3´) (*6*).

Amplicons were direct sequenced in an automated sequencer (ABI 377, Applied Biosystems, Foster City, CA, USA) using the Kit ABI PRISM Dye Terminator (Applied Biosystems) by the dideoxyribonucleotide chain terminator method (*11*). Nucleotide sequence obtained for the strain BeAn 626990 were compared with 44 other OROV N gene nucleotide sequences (AF164531–AF164558; AY704559–AY704568; AY993909–AY993912), and phylogenetic trees were constructed by using both neighbor-joining (NJ) (*12*) and maximum parsimony (MP) (*13*) methods implemented in the Mega 2.1 (*14*) and PAUP 4.0 software, respectively. Bootstrap analyses with 1,000 replicates were used to place confidence values on groupings (*15*).

Mice injected with the strain BeAn 626990 showed signs of disease 48–72 hours after intracerebral injection. By CF, the suspension prepared from the brain of infected mice showed positive reaction with the MIAF prepared for the Brazilian prototype strain of OROV BeAn 19991 (>32/16). No cross-reactivity was observed with any other MIAF prepared for the most common arboviruses currently circulating in the region. The monkey strain was then identified as OROV.

The N gene was found to be 693 nt in length, while the 5´ and 3´ terminal regions showed 290 and 161 nt, respectively. After the sequences assembly, accomplished by using the Seq Man program (DNA STAR software package, DNASTAR Inc., Madison, WI, USA), the full-length SRNA was determined (754 nt). The multiple sequence analysis for the entire N gene of the strain BeAn 626990, when Mega 2.1 software was used, showed homology with different OROV strains previously sequenced (92%–100%), as described (*6*).

Two overlapping open reading frames were determined. One consisted of 693 nt (231 amino acids) and was predicted to encode the N protein. The other open reading frame consisted of 273 nt (91 amino acids) and was predicted to encode the NSs proteins. Both coding regions were flanked by 2 noncoding regions, located at the 5´ and 3´ terminals that were 44 nt and 14 nt in length, respectively.

**Figure 2:**
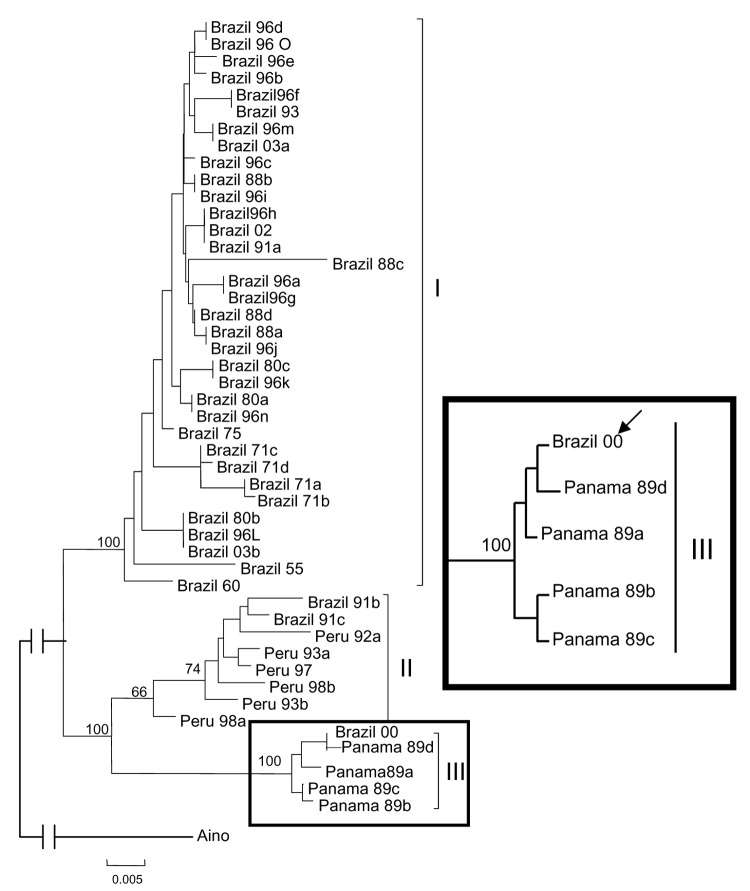
Phylogeny of Oropouche virus (OROV) strains isolated from different sources and periods by using the neighbor-joining and maximum parsimony methods. Bootstrap values were assigned over each internal branch nodes, and highest values were indicated by continuous arrows showing the presence of at least 3 lineages or genotypes (I, II, and III) of OROV. Bootstrap values for the 3 representative genotype clades are placed over each respective branch node. The black arrow indicates the position of the strain BeAn 626990 (Brazil 00) in the tree. The Aino N gene nucleotide sequence was used as an outgroup to root the tree. The scale bar represents 5% nucleotide sequence divergence.

Phylogenetic analysis carried out for 44 OROV N gene sequences (693 nt) with MP and NJ methods resulted in trees with similar topology, even though slightly low bootstrap values had been assigned for the MP consensus tree. The NJ tree suggested that the strain BeAn 626990 was more closely related to those included in genotype III. Bootstrap support values of 100%, 74%, and 100% were assigned for the genotypes I, II, and III, respectively (Figure 2).

## Conclusions

The known OROV epidemic sites are restricted to tropical areas of Central and South America, especially those in the Amazon Basin. From 1961 to 1980, in Brazil, OROV was reported in the northern state of Pará, where the most important epidemics occurred in Belém, and other regions of the state; hundred of thousands of persons were affected. From 1980 to 2004, OROV spread to 5 other northern Brazilian states (Amazonas, Amapá, Acre, Rondônia, and Tocantins) and 1 state in the northeast (Maranhão), indicating, in a short period of time, a dangerous epidemic potential (Table) (*5**,**6*).

**Table:** Examples of epidemics and isolates of Oropouche fever virus genetically characterized in Latin American countries, 1955–2004

Years of occurrence	Location	Genotypes
1955	Trinidad	I
	Brazil (Pará State)	
1960	Brazil (Santa Maria*)	I
1961, 1968, 1979–80	Brazil (Belém)	I
1967, 1979-80	Brazil (Bragança region)	
1972	Brazil (Baião)	
1974–75	Brazil (Santarém)	I
1988	Brazil (Tucuruí)	I
1996	Brazil (Oriximiná, Altamira)	I
2003	Brazil (Parauapebas)	I
2004	Brazil (Porto de Moz)	II
1992	Peru (Iquitos)	II
1998	Peru (Madre de Dios)	II
	Brazil (Acre State)	
1996	Brazil (Xapuri)	I
	Brazil (Amazonas State)	
1980–1981	Brazil (Manaus and Barcelos)	I
	Brazil (Maranhão State)	
1988	Brazil (Porto Franco)	I
	Brazil( Rondônia State)	
1991	Brazil (Ariquemes/Ouro Preto)	II
	Brazil (Tocantins State)	
1988	Brazil (Tocantinopolis)	I
2002	Brazil (Paranã)	I
1989	Panama†	III

*Bradypus tridactylus. †Chame, Chilibre, and San Miguelito.

The first isolation of OROV in the Arinos area in Minas Gerais, Brazil, is of concern because this virus, under favorable ecologic conditions, can spread, and Oropouche fever may develop in the local people, who are susceptible to OROV. In fact, explosive Oropouche fever epidemics have been reported in all of the following areas in Brazil: Maranhão, Tocantins, Amazonas, Acre, Pará, and Rondônia States. Such epidemics have also been reported in Iquitos and Madre de Dios, Peru; and in Bejuco, Panama (*5*).

Molecular studies (*6*) recognized 3 genotypes and suggested the distribution for OROV in South and Central America. The authors showed that in Brazil, genotype I, the most widespread in the country, and genotype II, found in Rondônia (a bordering state with Peru), and also in Para state (M.R.T. Nunes, unpub. data) cocirculate. In Peru, only genotype II has been identified. Genotype III has been reported only in Panama, whereas in Trinidad, only genotype I has been isolated (Table).

The genetic data obtained in this study, for the S segment of the strain BeAn 626990 (S: AY 117135), suggest its genetic relationship with other OROV strains. Phylogenetically, the monkey strain was shown to be more closely related to genotype III OROV isolates (Figure 2) and is the first report of genotype III in southeastern Brazil. More importantly, OROV was detected for the first time outside the known epidemiologic area for OROV transmission in South America. Our findings also represent the first report of OROV isolation from a monkey.

The potential impact of OROV in Brazil can be better assessed by viewing it in its demographic context. The southeast is the most populated region in Brazil, comprising urban areas such as the cities of Belo Horizonte, Rio de Janeiro, and São Paulo. The entire region has intense intra- and interregional migration, fostered by improved technology and transportation, which may lead to increases in the dissemination of both human and animal pathogens along these areas. Therefore, further studies on the ecology, epidemiology, and molecular epidemiology of OROV are needed, not only to improve knowledge about the epidemiology of this arbovirus but also to find out if its epidemic area is spreading outside the Amazon region and toward the southeast and other populated regions in Brazil. This situation is of particular concern because Oropouche fever epidemics have been reported in several small villages in the Brazilian Amazon region in 2003 and 2004 (Vasconcelos PFC, unpub. data). As a consequence, the risk of spreading OROV to susceptible areas has increased considerably in Brazil. More research and program development are needed to control this potential epidemic arboviral disease.

## References

[1] Büchen-Osmond C, ed. ICTVdB management. New York: Columbia University; 2003.

[2] Le Duc JW, Pinheiro FP. Oropouche fever. In: Monath TP, editor. The arboviruses: epidemiology and ecology. Boca Raton (FL): CRC Press; 1989. p.1–14.

[3] Anderson CR, Spence L, Downs WG, Aitken THG. Oropouche virus: a new human disease agent from Trinidad, West Indies. Am J Trop Med Hyg. 1961;10:574–8. 13683183 10.4269/ajtmh.1961.10.574

[4] Pinheiro FP, Pinheiro M, Bensabath G, Causey OR, Shope RE. Epidemia de vírus Oropouche em Belém. Revista do Servico Especial de Saúde Pública. 1962;12:15–23.

[5] Pinheiro FP, Travassos da Rosa APA, Vasconcelos PFC. Oropouche fever. In: Feigin RD, editor. Textbook of pediatric infectious diseases. Philadelphia: W.B. Saunders Co.; 2004. p. 2418–23.

[6] Saeed MF, Wang H, Nunes MRT, Vasconcelos PFC, Weaver SC, Shope RE, Nucleotide sequences and phylogeny of the nucleocapsid gene of Oropouche virus. J Gen Virol. 2000;81:743–8. 10675412 10.1099/0022-1317-81-3-743

[7] Wang H, Beasley DW, Li L, Holbrook MR, Barrett AD. Nucleotide sequence and deduced amino acid sequence of the medium RNA segment of Oropouche, a Simbu serogroup virus: comparison with the middle RNA of Bunyamwera and California serogroup viruses. Virus Res. 2001;73:153–62. 10.1016/S0168-1702(00)00234-3 11172919

[8] Aquino VH, Moreli ML, Moraes Figueiredo LT. Analysis of Oropouche virus L protein amino acid sequence showed the presence of an additional conserved region that could harbour an important role for the polymerase activity. Arch Virol. 2003;148:19–28. 10.1007/s00705-002-0913-4 12536293

[9] Vasconcelos PFC, Bryant JE, Travassos da Rosa APA, Tesh RB, Rodrigues SG, Barrett ADT. Genetic divergence and dispersal of yellow fever virus in Brazil. Emerg Infect Dis. 2004;10:1578–84. 15498159 10.3201/eid1009.040197 PMC3320275

[10] Shope RE, Sather GE. Arboviruses. In: Lennette EH, Schmidt NJ, editors. Diagnostic procedures for viral, rickettsial and chlamydial infections. 5th ed. Washington: American Public Health Association; 1979. p. 767–814.

[11] Sanger F, Nicklen S, Coulson AR. DNA sequencing with chain-terminating inhibitors. Proc Natl Acad Sci U S A. 1977;74:5463–7. 10.1073/pnas.74.12.5463 271968 PMC431765

[12] Saitou N, Nei M. The neighbor-joining method: a new method for reconstruction phylogenetic trees. Mol Biol Evol. 1987;4:406–25. 3447015 10.1093/oxfordjournals.molbev.a040454

[13] Swofford DL. PAUP. Phylogenetic analysis using parsimony (and other methods), version 4. Sunderland (MA): Sinauer Associates;1998.

[14] Kumar S, Tamura K, Nei M. Molecular evolutionary genetic analysis. Version 1.01. University Park (PA): Pennsylvania State University; 2000.

[15] Felsenstein J. Confidence limits on phylogenies: an approach using the bootstrap. Evolution. 1985;39:783–91. 10.2307/2408678 28561359

